# Nutrition Education and Community Pharmacy: A First Exploration of Current Attitudes and Practices in Northern Ireland

**DOI:** 10.3390/pharmacy7010027

**Published:** 2019-03-05

**Authors:** Pauline L. Douglas, Helen McCarthy, Lynn E. McCotter, Siobhan Gallen, Stephen McClean, Alison M. Gallagher, Sumantra Ray

**Affiliations:** 1 Nutrition Innovation Centre for Food and Health, Ulster University, Coleraine BT52 1SA, UK; gallen-s3@ulster.ac.uk (S.G.); AM.Gallagher@ulster.ac.uk (A.M.G.); 2 NNEdPro Global Centre for Nutrition and Health, Cambridge CB4 0WS, UK; helen.mccarthy1@vu.edu.au (H.M.); Lynn.McCotter@setrust.hscni.net (L.E.M.); s.ray@nnedpro.org.uk (S.R.); 3 College of Health and Biomedicine, Victoria University, Melbourne 3021, Australia; 4 School of Biomedical Sciences, Ulster University, Coleraine BT52 1SA, UK; s.mcclean@ulster.ac.uk

**Keywords:** nutritional education, community pharmacists, public health

## Abstract

Community pharmacist is one of the most prominent and accessible healthcare professions. The community pharmacists’ role in healthcare is evolving, with opportunities being taken to reduce pressure on primary care services. However, the question remains of how well community pharmacists are equipped for this changing role. This was a sequentially designed study using a mix of methods to explore nutrition education among community pharmacists in Northern Ireland. It consisted of two phases. Phase 1 was a cross-sectional exploration to map the attitudes and practice of Northern Ireland (NI) pharmacists towards diet-related health promotion and disease prevention. An online questionnaire with open and closed questions to gain both quantitative and qualitative responses was developed and distributed to community pharmacists practising in NI. A total of 91% considered nutrition important in reducing the global burden of disease. While the majority (89%) believed patients would value nutritional advice from a pharmacist, 74% were not confident in providing advice to a patient with diabetes. From the consensus gained in Phase 1 a nutrition education intervention (Phase 2) for pre-registration pharmacists was developed using the Hardens 10 question system. The training programme was advertised to pre-registration pharmacy students in NI. It was delivered by nutrition experts who have education qualifications. The intervention was evaluated using a before and after questionnaire that assessed knowledge, attitudes, and practice (KAP). Phase 2 did find sustained improvement from the baseline in KAP but there was a decline from immediately post-training to three months post-training. This suggests the need to further embed nutrition education. The education programme was found to be effective for the target population and sets the stage for the development of an implementation strategy for a wider roll-out with evaluation.

## 1. Introduction

Studies have shown that in a number of countries community pharmacists are up to four times more likely to interact with patients than the general practitioner (GP). In the United Kingdom GPs are often described as the ‘gatekeepers of primary care’; however, community pharmacists have an average of 12 to 15 consultations a year [1,2]. As members of one of the largest and most accessible healthcare professions, community-based pharmacists are ideally located to play a key role in disease prevention through education of the public about modifiable behaviours such as dietary intake (including safe use of nutritional supplements) and lifestyle decisions [3,4]. In addition, community pharmacies are often found in areas of deprivation, thereby allowing access to typically ‘hard to reach’ groups by being the first, and sometimes only, point of contact patients have with a healthcare professional [5].

In the United Kingdom, the Royal Pharmaceutical Society’s ‘Now or Never: shaping pharmacy for the future’ mandate has specified the need to expand the role of community pharmacists, highlighting the opportunity for them to reduce the pressure on GP services caused by long-term health conditions [6]. The public health challenges facing the UK include high blood pressure, tobacco use, harmful misuse of alcohol, high serum cholesterol, overweight, unhealthy diet and insufficient physical activity, all of which could, in principle, be addressed by community pharmacists as part of core healthcare [6]. However, the question remains as to how well community pharmacists are equipped for this changing role, particularly when, at present, nutrition and health promotion are not included in many pharmacy undergraduate curricula.

An increasing older population places further demands on health and social care, with the Institute of Public Health in Ireland projecting that the prevalence of long-term conditions among adults, such as hypertension, coronary heart disease, stroke and diabetes, will increase by 30% in Northern Ireland and 40% in the Republic of Ireland from 2007 to 2020 [7]. A focus on disease prevention is challenging given the existing pressure on service delivery and budget cuts. Nevertheless, pharmacists represent a unique, but hitherto underexplored, opportunity for the general public to access skilled diet and lifestyle advice in their local communities [8,9].

To date, no studies in the UK and Ireland have investigated nutrition in pharmacy education. In the small number of studies that exist worldwide, very positive outcomes were observed from educational interventions with undergraduate pharmacy students [9,10,11]. In one study, more than half of registered community pharmacists working in rural locations reported that their nutrition education was inadequate [12].

The previous literature is clear about what changes community pharmacies can make, but how these can be implemented still requires investigation [13]. A review by Brown et al. [14] concluded that there is good evidence to support community pharmacists engaging in public health and healthcare delivery services such as smoking cessation, cardiovascular disease prevention, hypertension, diabetes, asthma and heart failure, with weak but supporting evidence for weight management, sexual health, osteoporosis detection, substance abuse and chronic obstructive pulmonary disease.

A number of interventions have been undertaken using a range of educational techniques [15,16,17,18]. However, the novel aspect of the McNamara et al. study [16] was ascertaining the acceptability and utility of an educational package as a knowledge intervention tool.

The aims of this current study were to:

1. Undertake a preliminary mapping of Northern Ireland pharmacists’ attitudes and practices relating to diet-related health promotion and disease prevention.
2. Develop and pilot a nutrition education intervention for pre-registration pharmacists.

## 2. Materials and Methods

**Figure 1:**
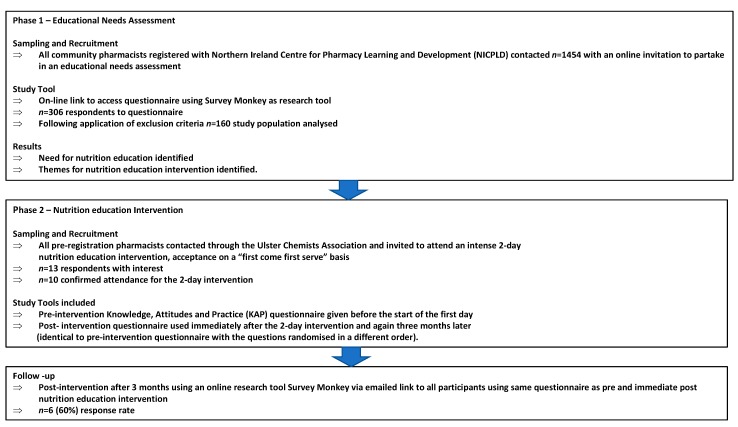
A sequentially designed study using a mix of methods to explore Nutrition Education in Community Pharmacists in Northern Ireland.

This study was sequentially designed using a mix of methods and was undertaken in 2 phases. (Figure 1). It was modelled, in part, on the Need for Nutrition Education/ Innovation Programme (NNEdPro) that continues to deliver nutrition training to medical students in the UK [19,20]. Four of the current researchers designed, delivered and evaluated the NNEdPro interventions. The educational work of NNEdPro continues to demonstrate the need for innovative ways to include nutrition in medical curricula to optimize patient outcomes. The questions now being asked are in relation to other healthcare workers who also have a role to play in health promotion and disease prevention.

Phase 1 was an educational needs assessment to gain consensus on a suitable nutrition education intervention. This intervention would be piloted and evaluated for pre-registration pharmacists in Phase 2 of the study.

### 2.1. Phase 1

#### 2.1.1. Study Design

Phase 1 adopted a mixed methods approach utilizing both qualitative and quantitative design.

The Phase 1 questionnaire was based on previous work by the authors [20]. It was adapted to a community pharmacist’s role [20] and reviewed by two registered pharmacists to ensure fitness for purpose.

SurveyMonkey was used as the online research tool. The questionnaire consisted of 23 questions that defined the demographic characteristics of the sample (*n* = 11) and investigated attitudes (*n* = 6) and practice (*n* = 6) of health promotion and disease prevention activities.

Demographic information gathered included personal (gender, if they trained in Northern Ireland, current registration status) and employment (hospital/community/other pharmacy role, urban/rural location) information. Respondent perception of the adequacy of any nutrition education (inadequate or adequate) they had received at undergraduate level, during pre-registration year or as a registered pharmacist was sought. Information regarding the last time they had engaged in nutrition education was also requested.

The questionnaire had both open and closed questions, facilitating both qualitative and quantitative evaluation.

The questions chosen were of a stand-alone nature and sufficiently generic so as not to require bespoke validation for this preliminary needs assessment. Each question offered a choice of four possible responses.

Attitude questions were on two themes:

1. Attitudes towards the importance of nutrition in public health;
2. Attitudes towards community pharmacists’ confidence in providing nutritional advice to patients.

Attitude and Practice (AP) questions were given a score by applying a Likert scale, with 1 being the most negative response and 4 the most positive (1–4). Total scores were calculated for each attitudinal theme and practice (Table A1).

#### 2.1.2. Recruitment

The Northern Ireland Centre for Pharmacy Learning and Development (NICPLD) is mostly concerned with the provision of materials for further education and continuing professional development of online or face-to-face events across Northern Ireland (https://www.nicpld.org). NICPLD hold a database (https://www.nicpld.org) of registered pharmacists who engage with lifelong learning and so were deemed an appropriate channel by which to engage with this grouping.

An online invitation to partake in the study was sent via a weblink e-mailed to registered pharmacists on the NICPLD database. This invited them to complete the questionnaire to participate in the survey assessing AP of practicing community pharmacists towards nutrition and health promotion and disease prevention activities. A reminder e-mail was distributed after one month. Social media sites including Facebook, Twitter and pharmacy forums were used to reach an extended audience.

#### 2.1.3. Data Collection

Respondents were self-selected. The responses to the online questionnaire were received by the researcher. All data received remained anonymous.

#### 2.1.4. Data Analysis

Questionnaires were excluded from analysis if they indicated the respondent was not a registered pharmacist practising in the community setting or not practising in Northern Ireland. Only complete questionnaires were included in the analysis.

Quantitative data analysis was conducted using SPSS for Windows (Version 21, SPSS Inc, Chicago, IL, USA) Descriptive statistical analysis was undertaken for demographic responses. Differences between education and population sub-groups were determined using a Chi-square test (or Fisher’s exact test when Chi-square assumptions were violated). Non-parametric tests were used to consider AP scores with medians (25th and 75th percentiles) reported. The Spearman Rank Order correlation coefficient was used to determine relationships between scores.

A qualitative content analysis as described by Morse and Field [21] was undertaken. Free text responses were read and reread and segments of the data were coded. Coded data were categorised to reflect the overall sense of the data and the relationships between categories. Related categories were then grouped into themes. Where appropriate, qualitative data were quantified by counting the number of times the same piece of information was reported in free text responses. These responses were used to identify and inform the teaching topics for Phase 2.

#### 2.1.5. Ethical Approval

Approval for the study was granted by the School of Biomedical Sciences Ethics Filter Committee, Ulster University (FCBMS-13-087). Consent was assumed if the respondent chose to complete the questionnaire.

### 2.2. Phase 2

#### 2.2.1. Study Design

From the results obtained in the Phase 1 needs assessment, the study design for Phase 2 was developed.

The intervention structure was based on a previous format that was used for a similar nutrition training intervention for medical students that was developed using Hardens 10 question system [20,22]. Topics and learning outcomes were adapted for pre-registration pharmacists and the community setting based on the outcome of Phase 1. The intervention consisted of a two-day workshop (Table A2) using a combination of lectures, demonstrations, simulations and interactive practical sessions that incorporated concepts of problem-based learning. Trainers included a medical doctor who is also a registered nutritionist, three registered dietitians, a pharmacist and an associate nutritionist. All trainers have a qualification in nutrition. All, with the exception of the pharmacist, have training and/or qualification(s) in education design and practice. The overall course aim was to educate future pharmacists about core principles of food and nutrition care in the community, especially related to nutritional screening, managing over- and undernutrition and systems-based nutritional management of health and disease. Key objectives included highlighting the role of a pharmacist in nutrition and health and providing a comprehensive overview of current national nutritional policy recommendations, as well as their practical application. A spiral learning approach revisited the topics on day 2 to build on the consolidation of basic concepts.

The previously described questionnaire for Phase 1 was adapted to also enable assessment of knowledge. Some of the demographic questions relating to years of practice and previous training were removed. The questionnaire for Phase 2 now consisted of 21 questions, including questions on demographics (*n* = 7), knowledge (*n* = 4), attitudes (*n* = 5) and practice (*n* = 5). It was administered before and after the education session and three months after the session to determine the Knowledge, Attitude and Practice (KAP) scores of the participants at the three time points. The three-month post-intervention questionnaire used SurveyMonkey as the online research tool.

#### 2.2.2. Recruitment

The Ulster Chemists’ Association is a non-profit organisation that promotes and protects community and primary care pharmacy across Northern Ireland. It is a membership body that is mainly concerned with community pharmacy practice. It was deemed a relevant body to facilitate recruitment to Phase 2.

A recruitment flyer advertising the course was distributed by the Ulster Chemist’s Association and advertised specifically for pre-registration pharmacists. Informed consent was assumed by signing up to the course and completion of the questionnaires.

#### 2.2.3. Data Collection

The questionnaire was administered before and after the education intervention session and three months after the session to determine Knowledge, Attitude and Practice (KAP) scores of the participants at the three time points.

#### 2.2.4. Data Analysis

Knowledge questions were scored using 1 for correct answers and a score of 0 for incorrect answers. Attitude and practice questions were scored using a Likert scale (1–4), where most negative responses were given a score of 1 and most positive scores were given a score of 4. Questionnaire items were randomised at each time point to minimise recall bias.

As the data were non-parametric, a Wilcoxon Signed Rank Test compared the total KAP scores between responses before and after the intervention. A longitudinal analysis of responses at the three-month follow-up was conducted using a Friedman Test.

#### 2.2.5. Ethical Approval

Ethical approval was granted by the Ulster University Research Ethics Filter Committee for Biomedical Sciences (FCBMS-14-035).

## 3. Results

### 3.1. Phase 1: Needs Assessment

**Figure 2:**
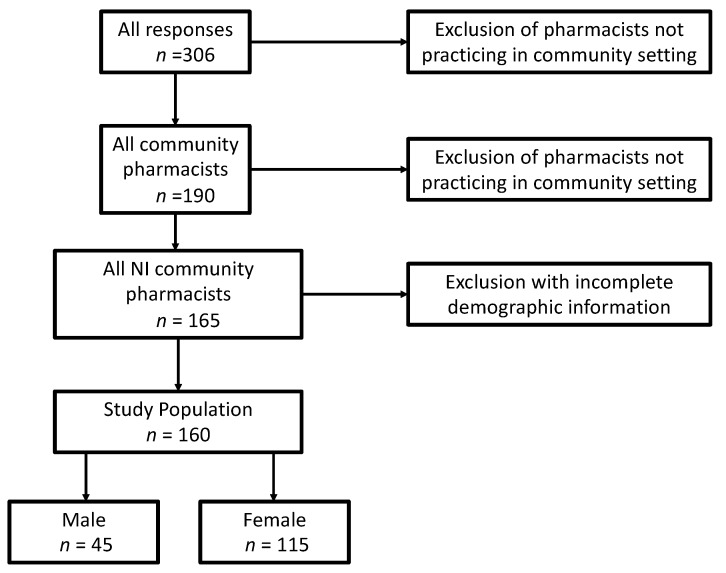
The number of initial respondents and the criteria required for inclusion in Phase 1 study analysis.

Three hundred and six questionnaires were completed and returned (a 15% response rate). After exclusion of those who did not meet the required inclusion criteria (Figure 2), 160 were eligible for analysis, providing a non-probability convenience sample size of 11% of registered community pharmacists in Northern Ireland.

Using the number of community pharmacists registered with the Pharmaceutical Society of Northern Ireland—1454 community pharmacists; 564 male and 890 female (2014)—the response rate to the questionnaire showed that a greater percentage of female community pharmacists responded to the questionnaire than their male counterparts (13% (*n* = 115) and 8% (*n* = 45), respectively). The characteristics of the participants are displayed in Table 1. When asked to consider the amount of nutrition education they had received at undergraduate (UG), pre-registration (PR) and professional (Prof) levels, approximately 80% (UG; 79%, PR; 83% and Prof; 81%) deemed this inadequate (Table 1). There was no significant difference between the perceived adequacy of training and the demographics: gender, location of undergraduate study or pre-registration year in the UK, number of years of practice or classification of the locality of NI employment (Table 1). A significant association was observed between the last time nutrition education was received and registration status (*P* = 0.003), with those registered for five years or fewer having received training most recently.

The scored results for attitudinal themes and practice are displayed in Table 2. Pharmacist attitudes towards nutrition in public health scored a median value of 11 (range: 11–12), while attitude regarding perceived ability to provide nutritional advice to patients (confidence) was 7 (range: 6–8). The median practice score value achieved was 9 (range: 8–10). Pharmacists’ confidence in their ability to provide nutritional advice had a stronger correlation with practice (*r* = 0.366) than attitudes towards the importance of nutrition in public health (*r* = 0.178).

Of the 160 pharmacists who participated, the majority (91%; *n* = 150) considered nutrition to be important in reducing the global burden of disease, 89% (*n* = 147) believed nutrition was important to a patient with digestive system complaints, and 87% (*n* = 143) believed patients would value nutritional advice from a pharmacist. In relation to perceived nutrition abilities, 74% (*n* = 122) of pharmacists considered nutrition problems to be managed inadequately in the community setting, 74% (*n* = 122) would not be confident if asked to provide dietary advice to a diabetic patient, and just over a quarter (25.6%; *n* = 42) reported that they would feel unprepared if asked to provide weight loss advice.

One respondent (0.6%) claimed they frequently advised clients about increasing their energy intake if required, 24% (*n* = 39) frequently advise patient groups about taking vitamin and mineral supplements, 33% (*n* = 54) often notified patients about potential food‒drug interactions, and 17% (*n* = 28) occasionally recommended the use of over-the-counter (OTC) calorific supplements that are not nutritionally complete. In the United Kingdom OTC are medicines or supplements that can be purchased from a pharmacy, shop or supermarket and do not require the person buying them to have visited a GP and received a medical prescription. One quarter (26%, *n* = 145) of pharmacists had never heard of the Malnutrition Universal Screening Tool (MUST).

Table 3 and Table 4 display themes and supportive quotes from free text responses in relation to the questions “How well do you feel nutritional problems are managed in the community?” and “Would you advise specific client groups about taking nutritional supplements? Please comment on the types of client groups and advice you would give”. The client groups most often advised about nutritional supplements were reported to be the elderly at 14% (*n* = 22), pregnant women at 13% (*n* = 21), and children at 8% (*n* = 13). The supplements most often mentioned in free text responses were vitamin D (*n* = 27), calcium (*n* = 14) and multivitamins (*n* = 10).

### 3.2. Phase 2 Pilot Nutrition Education Intervention

Ten pre-registered pharmacists who had all trained in Northern Ireland attended the education intervention (Table 5). Of these, 70% (*n* = 7) reported receiving no nutrition training or an inadequate amount during their undergraduate degree; 90% (*n* = 9) reported receiving no nutrition training or an inadequate amount during their pre-registration year.

All pre-registration pharmacists who participated in the pilot education session completed the KAP questionnaire before and after the education session. The overall KAP score significantly increased (*P* = 0.008) following the education session ((30.5; 4) versus (34.0; 2) for median; IQR respectively pre- versus post-). At the three-month follow-up 60% (*n* = 6) of questionnaires were completed. No significant difference (*P* = 0.142) in total KAP scores was found across the three time points (after three months: 31.5; 8).

All participants rated the session in terms of content and delivery as excellent (*n* = 7) or good (*n* = 3) with reasons such as “very informative” and “practical” provided in the comments box.

## 4. Discussion

A principal aim of this study was to develop and pilot a nutrition education intervention for pre-registration pharmacists. This also involved a preliminary mapping of attitudes and practices towards diet-related health promotion and disease prevention. Assessment of AP for community pharmacists confirmed a widespread feeling of inadequacy in relation to nutrition education. Furthermore, qualitative responses suggested that pharmacists considered health promotion and disease prevention activities to be within their professional role but recognised that they had an insufficient level of education. Based on findings from the AP questionnaire of community pharmacists, a pilot education session was developed, modelled on a previously successful programme for medical students [20]. The pharmacy pilot education session demonstrated a post-training improvement in knowledge. Embedded learning could not be conclusively demonstrated, although KAP remained higher than baseline at three months post-training.

### 4.1. Phase 1: Needs Assessment

The respondents exhibited a very positive attitudinal score towards nutrition and public health. This is encouraging given the expanding role of the pharmacy under national direction, as documented in Transforming Your Care [23], a Vision for pharmacy in the new NHS [24] and increased participation in community health screening [5]. However, concurrent with Ray et al. [20], confidence in ability appears to be a strong predictor of a higher practice score, i.e., engaging in health promotion activities with patients [11] achieved a statistically significant improvement in scores of self-efficacy following nutrition training, with students possessing an improved ability to communicate confidently with classmates. Just over one-fifth (21%) of this sample of pharmacists felt confident in providing dietary advice to a diabetic patient compared to 72% when presented with an overweight patient. This vast difference in confidence could be attributed to the training provided to pharmacists in preparation for the launch of OTC orlistat (Alli) in 2009.

One community pharmacist recognised this and commented, “When orlistat first became available OTC we were promoting heavily in pharmacy and giving excellent nutritional information along with the product … such as tips on healthy eating, recipe and meal ideas and advice on exercise”.

A study by Weidmann et al. [25] reported that 92% of pharmacists felt confident in their ability to supply the product and over two-thirds believed it provided a good opportunity to extend their role as a healthcare professional. As a result, pharmacists may be receptive to health promotion training, which could improve their confidence and competence in primary and early secondary prevention.

One example of disease prevention identified by the current study was recommending OTC oral nutritional supplements (ONS), particularly to older people and patients recuperating from an illness. Requests for the supply of these products could act as a timely opportunity for pharmacists to objectively assess the patient’s nutritional status using the ‘Malnutrition Universal Screening Tool’ (MUST) [26]. Patients classified as at risk of malnutrition have been shown to benefit more from a nutritional intervention than those rated as malnourished [27]. The majority (83%) of pharmacists have never heard of MUST. With appropriate training, the use of this screening tool could lead to more appropriate advice, such as food fortification or a timely referral to the GP or registered dietitian [28]. Additionally, the recent launch of MUST self-assessment is likely to result in more individuals seeking help and advice from their pharmacist in this area of practice [29].

The current study of community pharmacists has also highlighted concerns in relation to the use and supply of prescribed ONS. Qualitative responses implied that pharmacists are unhappy with the way malnutrition is managed in the community and have alluded to key areas where they think improvements can be made: compliance and follow-up. These two matters are the absolute core of ‘The pathway for using ONS in the management of malnutrition’ [30] and it appears pharmacists are observing obvious inadequacies with individual community pharmacists, reporting that they “don’t feel able to comment or advise” due to “inadequate training on these products”. Again, with appropriate training, pharmacists could reassess the need for prescribed ONS and discontinue treatment that is no longer required. Furthermore, patients with signs of poor compliance, returning unused supplements or complaining of the taste or texture, could be identified by pharmacists and the issues rectified (e.g., allow patient to taste other similar supplements) or refer patients back to the dietitian much earlier than the scheduled review.

The current study demonstrated that the elderly, pregnant women and children were the patient groups most likely to seek advice about vitamin supplements. This shows the broad spectrum of ‘healthy’ people pharmacists come into contact with on a daily basis, thereby providing a platform for increasing awareness and personalising the importance of good nutrition and lifestyle practices for the prevention of disease [31]. The responding community pharmacists listed the supplements that were most frequently observed in practice, including vitamin D, calcium and multivitamins. Of note, folic acid supplements were not listed as a vitamin the respondents would regularly recommend, despite highlighting pregnant women as a key population group. This is of topical interest due to the recent National Diet and Nutrition Survey 2015 [32]. In addition, update and refresher courses as part of lifelong learning may be of particular importance so the latest advice, such as folic acid recommendations, is not omitted.

The request for the sale of specific vitamin and mineral supplements provides opportunities for pharmacists to enquire about dietary intake and why they feel the need to supplement their diet, particularly when the patient has initiated the conversation. Revised Reference Nutrient Intakes in the United Kingdom [33] of 10 μg/d (400 IU/d) for those aged 4 y and above and for infants and younger children to achieve a safe intake in the range 8.5–10 μg/d (340–400 IU/d) at ages 0 to < 1 y and 10 μg/d (400 IU/d) at ages 1 to < 4 y also have implications for pharmacists.

Pharmacists also voiced concern over their source of nutrition information and find sales representatives’ information ‘biased’ for both ONS and infant nutrition. It appears that pharmacists want to learn from independent experts in the field and feel that “dietitians rarely reach out to educate other professionals and allow charlatan nutritionists to propagate nonsense”. In addition, pharmacists’ desire for interprofessional learning emerged as a core theme and supports the central focus of utilising Integrated Care Partnerships to create a collaborative network of care providers to coordinate local health and social care services [23]. Although dietitians are not specifically mentioned in this framework, the primary care team could benefit greatly from their expertise to equip primary care with the skills to instigate timely referrals and ensure a consistent dietary message is communicated to patients from all healthcare professionals [34].

### 4.2. Phase 2: Pilot Nutrition Education Intervention

The pilot education sessions demonstrated improvements in KAP scores from baseline. While there was some decrease in these scores between the end of the education session and three months post-training, the KAP was still higher than the baseline. The pilot was carried out with a small sample of pre-registration pharmacy students. The pre-registration year curriculum in Northern Ireland, includes ongoing professional training and information sessions on products such as infant milks as well as nutrition related to specific specialities. The Phase 2 study education sessions focused on key areas highlighted within the responses to Phase 1 questionnaire. These included diets in CVD and diabetes prevention and management, infant feeding, vitamin supplementation and malnutrition. Underpinning principles such as drug‒nutrient interactions were also included.

Previous research into the impact of nutritional training on pharmacists is limited and tends to focus specifically on the academic curriculum rather than the pre-registration year or post-registration. However, the studies that have been undertaken in this area suggest that, regardless of the training medium, improvements in nutritional knowledge and self-efficacy were demonstrated post-training [20,31,35,36].

For nutrition education to be effective it must result in embedded learning. This current study did find a sustained improvement from the baseline, but there was a decline in KAP from immediately post-training to three months post-training. This is reflective of similar findings in relation to other healthcare professionals [20].

To the authors’ knowledge, this is the first study in the UK to consider nutrition education in pharmacy. A small number of previous international studies offer educational interventions to undergraduate pharmacy students, a population that may not yet fully appreciate how applicable nutrition is to the community pharmacy setting [15,16]. Surveys involving health care providers are characterised by low and reduced response rates [37]. In this target population of busy professionals with varying work schedules and time constraints, an 11% response is comparable to other studies in this field, but the offer of a monetary reward may have improved this further [38]. The pattern of gender response observed (72% female) is similar to that reported in the literature [11,12].

Numbers of participants for the education session were low, reflecting the nature of this pilot study. One of the primary challenges of providing education sessions to busy professionals is the time commitment involved. While the benefits of face-to-face sessions and the ability to interact with others are clear, the practicality of this form of delivery needs to be considered prior to further roll-out of training. Having nutrition education embedded within the CPD training portfolio offered by the professional body could aid the face-to-face uptake; the provision of online “bite-size” training sessions should be explored and evaluated. The latter mode of delivery of training could be of particular benefit in terms of sustained and embedded learning as individuals could return to these on a regular basis to refresh learning.

An acknowledged limitation of this study is that the participants may have had a predisposed interest in nutrition. For example, some respondents stated, “unless you have a personal interest as I do, (nutrition) problems are not dealt with well” and “I have a keen personal interest in nutrition but few others have”. Despite this, it is also true to say that non-respondents may be, as yet, unaware of the importance nutrition has in their everyday role as Boggs et al. [12] reported that those pharmacists who felt they needed more training to answer patients’ nutrition-related queries were more likely to be those who had engaged in nutrition courses. Any increase in provision of nutrition education for this critical group could, in turn, create a desire for more information and encourage participation in continued professional development relating to nutrition.

Nutrition is a key aspect for consideration in every medical condition. Consequently, many healthcare professionals should have a foundational knowledge of nutrition to optimize patient care. Incorporation of nutrition education into curricula is a potential way of enhancing interprofessional learning [39].

## 5. Conclusions

This study has shown that Northern Ireland community pharmacists generally regard their nutrition education as inadequate, which is also reflected in practice. Current attitudes support the importance of nutrition in public health but a lack of confidence in this group is likely to affect nutritional practice in the community setting. This lack of confidence is likely associated with lower levels of self-efficacy due to knowledge and skills gaps, which in turn can be linked to education and training. Qualitative feedback strongly suggests that pharmacists would be receptive to educational intervention, particularly if it was delivered by independent nutritional experts within an interprofessional environment. Training in key topics identified by the survey suggested improvements in KAP of pre-registration community pharmacists. Utilisation of the unique access pharmacists have to patients of all ages and nutritional status has the potential to significantly improve nutrition practices in the primary care setting, leading to improved patient outcomes and more cost-effective treatments.

Based on the results of this paper, a stakeholder forum is planned as the next step to develop an implementation strategy on the education and training of pharmacists as well as the readiness of the nutrition education workforce to deliver such training in a sustainable fashion.

## Figures and Tables

**Table 1:** Demographic characteristics of the sample compared to perceived adequacy of education and the last time nutrition education was received.

	All *n* = 160	Nutrition Education in Undergraduate Degree *n* = 160			Nutrition Education in Pre-Registration Year *n* = 160			Nutrition Education as Qualified Pharmacist *n* = 160			The Last Time Nutrition Education Was Received (Years) *n* = 160			
		**Inadequate ^†^**	**Adequate ^†^**	***P***	**Inadequate ^†^**	**Adequate ^†^**	***P***	**Inadequate ^†^**	**Adequate ^†^**	***P***	**<2 ^†^**	**3–5 ^†^**	**>6 ^†^**	***P***
**Total**		126 (79)	34 (21)		132 (83)	28 (17)		130 (81)	30 (19)		58 (36)	66 (41)	36 (23)	
**Gender**														
Male	45 (28)	38 (30)	7 (21)		38 (29)	7 (25)		35 (27)	10 (33)		15 (26)	17 (26)	13 (36)	
Female	115 (72)	88 (70)	27 (79)	0.271	94 (71)	21 (75)	0.686	95 (73)	20 (67)	0.481	43 (74)	49 (74)	23 (64)	0.481
**Completed UG Degree in NI**														
Yes	128 (80)	100 (79)	28 (82)		104 (79)	24 (86)		107 (82)	21 (70)		50 (86)	52 (79)	26 (72)	
No	32 (20)	26 (21)	6 (18)	0.699	28 (21)	4 (14)	0.405	23 (18)	9 (30)	0.129	8 (14)	14 (21)	10 (28)	0.244
**Completed PR in NI**														
Yes	137 (86)	107 (85)	30 (88)		110 (83)	27 (96)		111 (85)	26 (87)		52 (90)	58 (88)	27 (75)	
No	23 (14)	19 (15)	4 (12)	0.786 ^‡^	22 (17)	1 (4)	0.081 ^‡^	19 (15)	4 (13)	1.0 ^‡^	6 (10)	8 (12)	9 (25)	0.114
**Registration Status**														
0–5 Years	55 (34)	42 (33)	13 (38)		42 (32)	13 (47)		48 (37)	7 (23)		27 (46)	25 (38)	3 (8)	
5–15 Years	51 (32)	36 (29)	15 (44)		40 (30)	11 (39)		39 (30)	12 (40)		16 (28)	21 (32)	14 (39)	
16+ Years	54 (34)	48 (38)	6 (18)	0.063	50 (38)	4 (14)	0.054	43 (33)	11 (37)	0.338	15 (26)	20 (30)	19 (53)	0.003 *
**Location of NI Employment ^§^**														
Rural	54 (34)	47 (37)	7 (21)		45 (34)	9 (32)		42 (32)	12 (40)		21 (36)	18 (27)	15 (42)	
Urban	101 (63)	76 (60)	25 (74)	0.084	83 (63)	18 (64)	0.857	83 (64)	18 (60)	0.509	36 (62)	47 (71)	18 (50)	0.202

Abbreviations: UG Undergraduate, PR Pre-Registration; Values compared using Chi-square test unless otherwise indicated; Statistical significance level of *P* < 0.05; †Data are *n* (%) corresponding to each column; ‡ Fishers exact test; § *n* = 155 for this question as five respondents answered ‘other’.

**Table 2:** Median scores for attitudinal themes and practice from Phase 1.

	Possible Score Range	Median Score (25th and 75th Percentiles)
Attitudinal Theme 1		
**Attitudes towards the importance of nutrition in public health (opinion)**	3–12	11 (11–12)
How important is nutrition in reducing the global burden of disease?		
How important is nutritional advice in patients with digestive complaints?		
Do you think patients would value nutritional advice from a pharmacist?		
**Attitudinal Theme 2**		
**Attitudes towards ability to provide nutritional advice (confidence)**	3–12	7 (6–8)
How well are nutritional problems managed in the community pharmacy setting?		
How confident are you in providing dietary advice to a diabetic?		
How equipped do you feel to offer weight loss advice?		
**Practice**	5–20	9 (8–10)
Advising increased energy intake when required due to medication side effects		
Advising patient about food‒drug interactions		
Advising specific patient groups about nutritional supplements (vitamins)		
Use of the Malnutrition Universal Screening Tool		
Recommendation of OTC supplements		

**Table 3:** Themes and supporting quotes from free text comments regarding the question “How well do you feel nutritional problems are managed in the community?” in Phase 1.

Theme Sub-Theme	Quotes
**Oral Nutrition Support (ONS)**	
Compliance & follow-up	“If patients dislike what they’re recommended they seem to simply become less compliant, rather than beginning a process of trial and error to find what’s suitable”. “I often ask patients if they take their nutritional supplements (e.g., Fortisip) and they often admit to not tolerating them and not being fully compliant”. “Insufficient review and follow-up, or when changes are made wastage of prescribed supplements when new ones introduced”. “Use of sip feeds and nutritional supplements is very expensive but seems to be poorly managed”. “Nurses and doctors do not really give advice apart from telling people to take a supplement, or just prescribe them Complan or Ensure and that’s it”. “Seems to be infrequent reviews and no follow up if patients actually use supplements appropriately”.
Full use of range	“Many products available with little guidance about differentiating between them. It would be more helpful if, like has been done for wound care there was a Northern Ireland formulary for dietary products”. “Always the same thing prescribed, e.g., Fortisip, without utilising the specialist versions of Compact, fibre, extra whenever they would be appropriate—unless overseen by secondary care”. “I feel there is a lack of awareness of products available, by patients and healthcare professionals”.
More training	“I don’t think pharmacists are able to advise or comment at all appropriate supplements for patients”. “Pharmacists are not given adequate training on these products”. “I know very little about PEG feeding but these patients and their families naturally still expect advice when they are lifting their prescription items”.
**Education** Interprofessional	“A lot of the blame lies with dieticians. They rarely reach out to educate other professionals and allow charlatan nutritionists to propagate nonsense”. “Due to insufficient knowledge base and poor, if any, contact between community dietician team and pharmacist”. “GPs and community pharmacists need to work together to promote good nutrition, weight loss and a healthy lifestyle and diet”. “Not enough emphasis on the information and advice pharmacists could provide for patients”.
Bias	“I would be concerned about evidence for certain baby milk prescribed by GPs; it seems to me that the company reps decide what patients get—not the independent experts”. “Infant nutrition advice is limited as the training provided is usually by the formula milk companies so is biased”.
More training	“Lack of knowledge”. “Feel it is an important area we can recommend for health promotion but confidence in our knowledge is lacking”. “I always refer to a dietician as I do not have sufficient knowledge to advise”. “Neither doctors or pharmacists are properly trained on nutrition”. “I think there is a lot of education to be done and that poor nutrition is contributing to ill health in many of our patients”. “I believe many health professionals are missing opportunities to intervene in patient nutrition”. “Nutritional problems need to be addressed more proactively. Pharmacy needs to step up a gear to accommodate the public desire for more evidence-based education”.

**Table 4:** The frequency of recurring themes in response to the question in Phase 1, “Would you advise specific client groups about taking nutritional supplements? Please comment on the types of client groups and advice you would give”.

Types of Client Groups	*n*	Vitamins/Supplements Mentioned	*n*
Elderly	22	Vitamin D	27
Pregnancy	21	Calcium	14
Children	13	Multivitamins/tonics	10
Teenagers/young females/childbearing age	8	Omega/fish oil	7
Illness/post-illness	8	Glucosamine	5
Housebound/little sunlight	5	Vitamin C	5
		Iron	4

*n* = number of recorded repetitions of theme found within free text responses.

**Table 5:** Characteristics of the pre-registration pharmacists participating in the education sessions (Phase 2).

Characteristic	*n* (%)
Male:female	1 (10): 9 (90)
Hospital:community pharmacist	1 (10): 9 (90)
Rural:urban pharmacy	2 (20): 8 (80)

## Appendix A

**Table A1:** Response to Knowledge, Attitude and Practice (KAP) questionnaire pre- and post- pilot education sessions.

Question	Response Options	Pre- (*n* = 10)	Post- (*n* = 10)	Three Months After (*n* = 6)
How well do you think nutrition problems are managed in the community? (A)	Badly	1	0	0
	Inadequately	5	9	4
	Adequately	4	1	1
	Very well	0	0	1
Is there good evidence to suggest that fish oil consumption (omega-3 fatty acids) is helpful in the management of: (K)				
Respiratory disease?	Yes	1	8	5
Cardiovascular disease?	Yes	10	10	6
Gastrointestinal disease?	Yes	2	7	6
Endocrine disease?	Yes	2	8	6
How confident are you in recommending dietary patterns for clients with diabetes? (P)	Not confident	4	0	0
	Somewhat confident	5	1	1
	Confident	1	7	4
	Very confident	0	2	1
What key nutrient would you recommend that weaning babies particularly require? (K)	Vitamin C	3	0	1
	Calcium	2	6	2
	Iron	4	3	3
	Sodium	1	1	0
Would you feel equipped to give general nutritional advice to clients where appropriate or required? (A)	Not at all	2	0	0
	Inadequately	6	0	1
	Adequately	1	9	4
	Very well	1	1	1
Have you ever advised a client on how to increase their energy intake when prescribed long-term use of a medication that has an anorexic side effect? (P)	Never	10	9	5
	Occasionally	0	1	0
	Frequently	0	0	1
	All the time	0	0	0
Do you think that, from a public health perspective, nutrition is important in reducing the global burden of disease? (A)	Not at all	0	0	0
	Not much	1	0	0
	Somewhat	2	1	0
	Very much	7	9	6
Which micronutrient deficiency should you be aware of in people of Southeast Asian origin? (K)	Vitamin A	3	1	4
	Iodine	2	2	0
	Vitamin D	1	7	1
	Zinc	4	0	1
Would you advise clients about food‒drug interactions, such as advising clients prescribed statins or benzodiazepines to avoid grapefruit juice? (P)	Never	3	0	1
	Occasionally	6	1	4
	Frequently	0	6	1
	All the time	1	3	0
Have you discussed with clients what specialist gluten-free products they would like to see available in the pharmacy? (P)	Never	7	6	3
	Occasionally	2	4	1
	Frequently	1	0	2
	All the time	0	0	0
Would you advise specific client groups about taking nutritional supplements? (P)	Never	4	1	0
	Occasionally	4	6	5
	Frequently	2	3	1
	All the time	0	0	0
When should the MUST for adults be used in the community or care home settings? (K)	Never	0	0	0
	Occasionally	4	4	1
	Frequently	4	2	1
	All the time	2	4	4
How important do you think nutritional advice is in clients with conditions affecting the digestive system? (A)	Not at all	0	0	0
	Not much	0	0	0
	Somewhat	1	1	1
	Very much	9	9	5
Do you think clients would value general nutritional advice from a pharmacist? (A)	Not at all	0	0	0
	Not much	0	0	0
	Somewhat	6	3	1
	Very much	4	7	5

* It should be noted that, for the attitude and practice questions, there will not be a correct answer.

**Table A2:** Two-day workshop programme.

Day 1	
09.00	Registration for Day 1
09.30	Welcome & Introduction SR & PD
09.40	Pre-questionnaire
10.00	Nutrition LMcC
10.35	Nutrition, public health and policy: A pharmacist’s responsibility SR
11.15	Coffee break
11.40	Nutrition and cardiovascular disease SR & CL
12.30	Best practice nutrition advice for baby and new mums HMcC
13.00	Lunch
14.00	Mini problem-based learning stations (25 min each) All team MUST/STAMP Reputable sources for pharmacists to obtain/direct clients for nutritional advice Texture modification Baby and new mum Nutritional products
16.15	Coffee
16.30	Feedback by students
17.00	Sum Up
17.15	Close of Day 1
**Day 2**	
09.00	Registration for Day 2
09.20	Nutrition and metabolic/endocrine disease CL & SR
09.50	Nutrition and gastrointestinal diseases HMcC & LMcC
10.20	Nutrition and respiratory diseases PD
10.50	Coffee break
11.10	Drug and nutrient/food interactions SG
11.40	Vitamin and mineral supplements: what’s the evidence? SG
12.10	Mini problem-based learning stations All team
13.00	Lunch
14.00	Mini problem-based learning stations All team
14.25	Feedback by students
15.00	Post-questionnaire
15.20	Coffee
15.35	Wrap up
16.00	Certificates
16.15	Close of Day 2

SR—Licensed Medical doctor, Registered Public Health Nutritionist with special interests in Cardiovascular Disease Prevention and Medical Nutrition Education, Course Director in Nutrition Science at the University of Cambridge Institute of Continuing Education, Executive Director NNEdPro Global Centre for Nutrition and Health, Cambridge.

PD—Registered Dietitian, Course Director BSc Hons Human Nutrition at Nutrition Innovation Centre for Food and Health, Ulster University, Fellow of the Higher Education Academy, Clinical Dietetics Facilitator, Education Director NNEdPro Global Centre for Nutrition and Health, Cambridge.

LMcC—Registered Dietitian with teaching experience, NNEdPro Coordinator.

CL—Registered Public Health Nutritionist and Fellow of the Higher Education Academy, Doctorate working in hospital malnutrition, piloting the implementation of the Integrated Nutrition Pathway for Acute Care in five hospitals across Canada.

HMcC—RD, Fellow of the Higher Education Academy, paediatric expert, Course Lead Bachelor of Human Nutrition, Victoria University Melbourne, Australia.

SG—Registered Pharmacist, Master’s qualification in Human Nutrition.

The two-day programme consisted of a combination of lectures, demonstrations, simulations and interactive practical sessions that each incorporated concepts of problem-based learning. Various modes of teaching were used in an integrative manner throughout the two-day workshop and throughout the individual elements.
